# Combined Spinal-Epidural Analgesia for Laboring Parturient with Arnold-Chiari Type I Malformation: A Case Report and a Review of the Literature

**DOI:** 10.1155/2013/512915

**Published:** 2013-03-27

**Authors:** Clark K. Choi, Kalpana Tyagaraj

**Affiliations:** Department of Anesthesiology, Maimonides Medical Center, 4802 10th Avenue, Brooklyn, NY 11219, USA

## Abstract

Anesthetic management of laboring parturients with Arnold-Chiari type I malformation poses a difficult challenge for the anesthesiologist. The increase in intracranial pressure during uterine contractions, coughing, valsalva maneuvers, and expulsion of the fetus can be detrimental to the mother during the process of labor and delivery. No concrete evidence has implicated high cerebral spinal fluid pressure on maternal and fetal complications. The literature on the use of neuraxial techniques for managing parturients with Arnold-Chiari is extremely scarce. While most anesthesiologists advocate epidural analgesia for management of labor pain and spinal anesthesia for cesarean section, we are the first to report the use of combined spinal-epidural analgesia for managing labor pain in a pregnant woman with Arnold-Chiari type I malformation. Also, we have reviewed the literature and presented information from case reports and case series to support the safe usage of neuraxial techniques in these patients.

## 1. Introduction

Arnold-Chiari type I malformation (ACM-I) is a congenital neurological anomaly associated with prolapse of the cerebellar tonsils into the magnum foramen [1, 2]. Approximately 30% to 50% of the ACM-I patients have associated syringomyelia. Incidence of ACM-I ranges between 0.56% and 0.77% on magnetic resonance imaging (MRI) studies, of which 15% to 30% are asymptomatic. This abnormality is mostly predisposed to women, with a female-to-male ratio of 3 : 1. Symptoms including headaches, neck and shoulder pain, paresthesia, loss of pain and temperature sensation in the upper extremities, and unsteady gait are the usual manifestations seen during early adolescence into adulthood. Severity of the symptoms ranges from mild when tonsillar herniation is larger than 5 mm to severe if it is more than 12 mm on the sagittal MRI view [3].

A combined spinal-epidural (CSE) technique was used to provide labor analgesia for our parturient with ACM-I. We also conducted a literature search for our case presentation using a public accessible medical database MEDLINE. Individual key words were entered into the query: “Arnold-Chiari,” “vaginal delivery,” “pregnancy,” “combined spinal-epidural analgesia,” “epidural analgesia,” “spinal analgesia,” “cesarean section,” “perioperative outcomes,” and their combinations. Only articles in English language were selected. The database search yielded limited number of articles, mainly case reports and case series (Table 1).

## 2. Case Presentation

**Figure 1:**
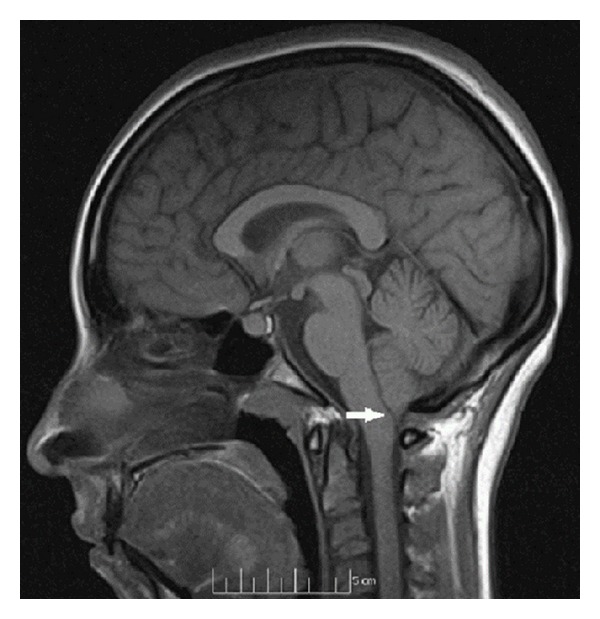
Sagittal magnetic resonance image of Arnold-Chiari type I malformation. White arrow denotes the 7 mm tonsillar herniation from the cerebellum. No syringomyelia is seen.

A 17-year-old female, G1P0, with history of hypothyroidism and ACM-I diagnosed during childhood, presented with symptoms of occasional headache and neck pain. She denied any visual disturbances or abnormal pain and temperature sensation in both upper extremities. She was consulted by a multidisciplinary team, including the anesthesiologist, perinatologist, and neurologist, for a planned labor induction with instrument-assisted vaginal delivery. MRI of the brain showed a 7 mm cerebellar tonsil herniation into the foramen magnum without syringomyelia (Figure 1).

Physical examination showed a 62 kg afebrile woman, in mild distress from uterine contractions, with a blood pressure of 134/89 mmHg, pulse of 62/min, respiratory rate of 12/min, and pulse oximetry saturation of 99%. Baseline laboratory values were hemoglobin 11.9 g/dL and platelets 206 × 10^9^/L. With a single attempt, CSE was achieved using a 17-gauge Tuohy needle and a 5-inch 27-gauge Whitacre spinal needle at the midline of the L3-L4 interspinous space while the patient was in a sitting position. Analgesia was obtained with fentanyl 15 *μ*g and bupivacaine 1.5 mg intrathecally. Aspiration of the epidural catheter and test dose of lidocaine 1.5% with epinephrine 1 : 200,000 were negative. A continuous epidural infusion of bupivacaine 0.1% and fentanyl 0.0002% was initiated at the rate of 10 mL/h. A 5 mL bolus of bupivacaine 0.25% was injected epidurally 90 minutes before the onset of fetal expulsion and subsequently augmented with another bolus to provide a denser block to minimize the urge to push. 

Fetal heart rate (FHR) and uterine contractions were continuously monitored by an external cardiotocograph. Category I FHR tracing was noted throughout the first and second stages of labor. Maternal and fetal hemodynamics were stable during the entire labor and delivery process. Labor progressed smoothly and lasted for 9 hours. The patient gave birth to a 2,995 g healthy girl using vacuum-assisted extraction. Apgar scores at 1 and 5 minutes were 9 and 9, respectively. Estimated blood loss was 200 mL. The patient had an uneventful postpartum course without any neurological sequelae. She was discharged home three days later. 

## 3. Discussion

Attempts to demonstrate the efficacy and safety of neuraxial technique (epidural versus spinal) in a pregnant woman with ACM-I have been the subject of controversy. The risks of accidental dural puncture with the epidural needle can lead to tentorial herniation, decreased cerebral perfusion pressure, and brain shifts. Intentional intrathecal puncture using spinal needle can also present with similar manifestations but the magnitude of the effect and incidence is relatively less than the epidural needle-induced dural puncture due to the larger size of the dural puncture. Selection of smaller size epidural and spinal needles is an important factor to improve safety, but, ultimately, the danger can be significantly minimized with an experienced and trained anesthesiologist to avoid inadvertent dural puncture as well as multiple needle attempts.

The safety of providing intrathecal analgesia for immediate pain relief during labor and anesthesia for cesarean section (CS) can be effectively implemented provided that there are no acute worsening of clinical signs and symptoms of intracranial pressure (ICP). In our case presentation, we selected the use of CSE to provide immediate pain relief intrathecally for our patient and the epidural catheter to administer intermittent extradural boluses for analgesia during the course of labor and delivery as well as for anesthesia for emergent CS due to obstetrical and fetal concerns. Had our patient developed severe or new onset of neurological symptoms during pregnancy, neuraxial technique would be contraindicated. Even without any absolute contraindications, there are currently no firm guidelines to suggest preference for general anesthesia over neuraxial techniques except many believe that the patients with ACM-I have inherent high ICP; therefore, neuraxial techniques are unsuitable choice for analgesia and anesthesia [12–17]. General anesthesia is not without any risks as airway management by rapid sequence induction and intubation from direct laryngoscopy to protect parturients from aspiration can potentially increase ICP. Difficult intubation, as encountered in some of the obese pregnant patients, can cause rapid desaturation leading to hypoxia and hypercarbia which further enhance the effect on ICP. Landau et al. described a case of successful spinal anesthesia after surgical decompression of a parturient with ACM-I [3]. Moreover, spinal anesthesia for CS has been successfully performed for undiagnosed parturients with ACM-I and also those without neurosurgery [4–6].

The choice for the mode of delivery (vaginal versus CS) is also a controversial issue. The contractile force of the uterus on cerebral spinal fluid (CSF) can cause an increase in ICP and unsuspected herniation. The hydrodynamic effect on CSF pressure during labor was investigated by several researchers in the 1960s [18–21]. Changes in the intra-abdominal and intrathoracic pressure secondary to sensation of pain were factors causing elevated CSF pressure during uterine contractions. Pain can induce elevated CSF but whether it contributes to a significant impact on unfavorable maternal and fetal outcomes is unclear. Mueller and Oró reported three case presentations of normal spontaneous vaginal delivery in parturients with ACM-I without receiving epidural block during labor [7]. Semple and McClure [8] and Nel et al. [9] used epidural anesthesia for CS without a clear obstetric indication other than the fear of increased ICP from straining in the second stage of delivery except from one case report described by Parker et al. [10]. Newhouse et al. managed successfully a parturient with ACM-I and sickle cell disease presented with acute pain crisis using epidural analgesia via vacuum-assisted vaginal delivery without neurological complications [11].

Key points in the anesthetic management of laboring parturients with ACM-I include (1) early CSE analgesia to decrease painful uterine contractions to limit intra-abdominal and intrathoracic excursions to dampen elevated CSF pressure; (2) slow titration of bolus through the epidural to prevent undue extradural pressure; (3) vacuum-assisted vaginal delivery in the second stage of labor to minimize increase in ICP during fetal expulsion and maternal valsalva maneuvers; and (4) minimization of wide variations of maternal hemodynamics to preserve adequate cerebral perfusion pressure. 

In summary, CSE labor analgesia can provide safe and effective pain relief to parturient with ACM-I. We emphasize the importance of multidisciplinary approach to tailor an individualized care plan for favorable maternal and fetal outcomes.

## Figures and Tables

**Table 1:** Summary of anesthetic management of patients with Arnold-Chiari type I malformation.

Authors and references	Age (years)	Symptoms	Tonsillar herniation (mm)	Syrinx	Surgery before labor	Gravida and para	Gestation age (wks)	Delivery method	Apgar at 1 min and 5 min	Neuraxial method	Maternal postpartum symptoms
Landau et al. [3]	31	Headache, vertigo, nausea, nystagmus, lower extremity, and hyperreflexia	Descended to C3	No	Yes	G2P1	37	CS	9, 10	Spinal	No change

	30	Arm and leg tingling	Not reported	No	No	G1P0	Not reported	CS	Not reported**	Continuous spinal	PDPH requiring blood patch
	32	Arm and leg tingling	Not reported	No	No	G2P1	Not reported	CS	Not reported**	Spinal	No change
	35	Arm and leg tingling and headache	Not reported	No	No	G3P2	Not reported	CS	Not reported**	Spinal	No change
	20	None	Undiagnosed	Undiagnosed	No	G1P0	Not reported	NSVD	Not reported**	Epidural	No change
Chantigian et al. [4]	39	Headache, right arm paresthesia, and numbness	Not reported	No	No	G3P2	Not reported	NSVD	Not reported**	Epidural	No change
	21	Headache	Not reported	No	No	G2P1	Not reported	NSVD	Not reported**	Epidural	No change
	25	None	Undiagnosed	Undiagnosed	No	G1P0	Not reported	NSVD	Not reported**	Epidural	No change
	21	None	Undiagnosed	Undiagnosed	No	G1P0	Not reported	NSVD	Not reported**	Epidural	No change
	25	None	Undiagnosed	Undiagnosed	No	G2P1	Not reported	NSVD	Not reported**	Epidural	No change

Kuczkowski [5]	35	Headache, vertigo, and upper extremity paresthesia	Not reported	No	No	G1P0	37	CS	Not reported**	Spinal	No change

Hullander et al. [6]	31	None	Undiagnosed	Undiagnosed	No	G1P0	Not reported	CS	Not reported**	Epidural and spinal	Headache and neck pain requiring blood patch

	30	Headache, dizziness, vision changes, upper extremities paresthesia, and dyspnea	8	Yes	No	G2P1	32	NSVD	Not reported*	Epidural	No change
Mueller and Oró [7]	27	Headache, tinnitus, and dizziness	4	No	Yes	G2P1	Not reported	NSVD	Not reported**	Epidural	No change
	31	Headache, blurred vision, hoarseness, dizziness, neck pain, upper extremities paresthesia, tinnitus, and dyspnea	10	No	Yes	Not reported	Not reported	NSVD	Not reported**	Epidural	No change
	32	Headache, neck pain, dizziness, hoarseness, dysphagia, and upper extremity paresthesia	13	No	Yes	G1P0	Not reported	NSVD	Not reported**	Epidural	Neck pain, spasm

Semple and McClure [8]	30	Ataxia, upper extremity paresthesia, and preeclamptic	Not reported	No	No	Not reported	30	CS	8, 9	Epidural	No change

Nel et al. [9]	31	Headache, reduced pain and temperature, and wasting on left hand	Not reported	Yes	No	G2P1	38	CS	Not reported**	Epidural	No change

Parker et al. [10]	26	Headaches, peripheral paresthesias, and weakness	Not reported	Yes	No	G1P0	38	CS	Not reported**	Epidural	No change
	30	None	Not reported	Yes	No	G1P0	39	CS	Not reported**	Epidural	No change

Newhouse and Kuczkowski [11]	20	Headache, chest pain, and hand and foot numbness	Not reported	Yes	No	G1P0	35	NSVD	8, 9	Epidural	No change

Our patient	17	Headache and neck pain	7	No	No	G1P0	39	NSVD	9, 9	CSE	No change

NSVD: normal spontaneous vaginal delivery; CS: cesarean section; CSE: combined spinal-epidural; PDPH: postdural puncture headache. *Neonate required hospitalization for pneumonia and respiratory distress. **Healthy neonate.
